# *Eukaryotic translation initiation factor 4E binding protein 1* (*EIF4EBP1*) expression in glioblastoma is driven by ETS1- and MYBL2-dependent transcriptional activation

**DOI:** 10.1038/s41420-022-00883-z

**Published:** 2022-02-28

**Authors:** Laura Hauffe, Daniel Picard, Julian Musa, Marc Remke, Thomas G. P. Grünewald, Barak Rotblat, Guido Reifenberger, Gabriel Leprivier

**Affiliations:** 1 Institute of Neuropathology, Medical Faculty, University Hospital Düsseldorf, Heinrich Heine University, Düsseldorf, Germany; 2 Department of Pediatric Oncology, Hematology, and Clinical Immunology, Medical Faculty, University Hospital Düsseldorf, Heinrich Heine University, Düsseldorf, Germany; 3 German Cancer Consortium (DKTK), partner site Essen/Düsseldorf, Düsseldorf, Germany; 4 Max-Eder Research Group for Pediatric Sarcoma Biology, Institute of Pathology, Faculty of Medicine, LMU Munich, Munich, Germany; 5 Division of Translational Pediatric Sarcoma Research, German Cancer Research Center (DKFZ), Heidelberg, Germany; 6 Hopp Children’s Cancer Center (KiTZ), Heidelberg, Germany; 7 Department of General Visceral and Transplantation Surgery, Heidelberg University Hospital, Heidelberg, Germany; 8 Institute of Pathology, Heidelberg University Hospital, Heidelberg, Germany; 9 Department of Life Sciences, Ben-Gurion University of the Negev, Beer Sheva, Israel; 10 The National Institute for Biotechnology in the Negev, Beer Sheva, Israel

**Keywords:** CNS cancer, Transcription

## Abstract

*Eukaryotic translation initiation factor 4E binding protein 1* (*EIF4EBP1*) encodes the 4EBP1 protein, a negative regulator of mRNA translation and a substrate of the mechanistic target of rapamycin (mTOR), whose function and relevance in cancer is still under debate. Here, we analyzed *EIF4EBP1* expression in different glioma patient cohorts and investigated its mode of transcriptional regulation in glioblastoma cells. We verified that *EIF4EBP1* mRNA is overexpressed in malignant gliomas, including isocitrate dehydrogenase (IDH)-wildtype glioblastomas, relative to non-neoplastic brain tissue in multiple publically available datasets. Our analyses revealed that *EIF4EBP1* overexpression in malignant gliomas is neither due to gene amplification nor to altered DNA methylation, but rather results from aberrant transcriptional activation by distinct transcription factors. We found seven transcription factor candidates co-expressed with *EIF4EBP1* in gliomas and bound to the *EIF4EBP1* promoter, as revealed by chromatin immunoprecipitation (ChIP)-sequencing data. We investigated the ability of these candidates to activate the *EIF4EBP1* promoter using luciferase reporter assays, which supported four transcription factors as candidate *EIF4EBP1* regulators, namely MYBL2, ETS1, HIF-1A, and E2F6. Finally, by employing transient knock-down experiments to repress either of these transcription factors, we identified MYBL2 and ETS1 as the relevant transcriptional drivers of enhanced *EIF4EBP1* expression in malignant glioma cells. Taken together, our findings confirm enhanced expression of *EIF4EBP1* in malignant gliomas relative to non-neoplastic brain tissue and characterize the underlying molecular pathomechanisms.

## Introduction

Eukaryotic initiation factor 4E binding protein 1 (*EIF4EBP1*) encodes 4EBP1, a substrate of the nutrient-responsive hub mechanistic target of rapamycin complex 1 (mTORC1). Upon nutrient deprivation, 4EBP1 gets activated [1] and in turn inhibits mRNA translation initiation by binding the mRNA cap-binding protein eIF4E [2]. The role of 4EBP1 in cancer is still being debated, as 4EBP1 exhibits both tumor-suppressive [3–6] and pro-tumorigenic functions [7, 8], depending on the tumor types. Accordingly, the clinical relevance of *EIF4EBP1* expression is strongly dependent on the tumor entity. On the one hand, loss of *EIF4EBP1* and low 4EBP1 levels have been linked to poor survival of patients with head and neck squamous cell carcinoma [3] or prostate cancer [9]. On the other hand, *EIF4EBP1*, as part of the 8p11-12 amplicon, is frequently amplified in breast cancer [10, 11]. Furthermore, high *EIF4EBP1* levels are associated with poor survival in all The Cancer Genome Atlas (TCGA) cancer entities combined [12], as well as in breast cancer [10, 11], colorectal cancer [13], hepatocellular carcinoma [14] or diffuse large B-cell lymphoma [15]. However, the prognostic relevance of *EIF4EBP1* expression in other individual tumor entities is poorly established, and the mechanisms regulating *EIF4EBP1* expression in distinct types of cancer warrant further investigations.

To date, only a few transcription factors have been characterized to bind the *EIF4EBP1* promoter and stimulate *EIF4EBP1* transcription in normal and cancer cells. These include the MYC oncoprotein [15], the androgen receptor [16], the stress response regulators ATF4 [15] and ATF5 [17], as well as HIF-1A [18]. In particular, MYC and ATF4 have been shown to co-regulate *EIF4EBP1* transcription in cancer cells [15], providing one potential mechanism underlying *EIF4EBP1* overexpression in cancer. The possible involvement of yet other transcription factors in regulating *EIF4EBP1* expression in human cancers remains to be investigated.

Glioblastoma is the most common and most malignant primary glial tumor type of the central nervous system (CNS) that according to the World Health Organization (WHO) classification of CNS tumors corresponds to CNS WHO grade 4 [19, 20]. This tumor entity nowadays comprises only isocitrate dehydrogenase (IDH)-wildtype tumors [20], as opposed to the previous WHO classification of CNS tumors which also included IDH-mutant tumors [19]. IDH-wildtype glioblastomas are diffuse astrocytic gliomas that grow invasively in the brain parenchyma, are highly proliferative and angiogenic, and are characterized by the presence of hypoxic and necrotic regions [21]. Median survival time is only around 15 months after diagnosis [19], despite standard of care treatment [22, 23]. The initiation and progression of IDH-wildtype glioblastomas are driven by genetic alterations that inactivate tumor suppressor genes like *PTEN*, *CDKN2A*, *RB1*, *NF1*, and *TP53*, or activate cellular oncogenes like *EGFR*, *PDGFRA*, *CDK4*, *MDM2*, and *PIK3CA* [24]. In addition, epigenetic changes and alteration of transcription factor-driven gene expression contribute to glioblastoma pathogenesis [25].

Using different publically available malignant glioma datasets and chromatin immunoprecipitation (ChIP)-sequencing data, we confirmed that *EIF4EBP1* mRNA expression is elevated in malignant glioma tissues, relative to non-neoplastic brain tissue, and identified seven transcription factor candidates supporting *EIF4EBP1* overexpression. We showed with promoter-reporter assays and genetic knockdown experiments that among these factors, ETS1 and MYBL2 regulate *EIF4EBP1* transcription in IDH-wildtype glioblastoma cells.

## Results

### *EIF4EBP1* mRNA levels in malignant gliomas are elevated independently of gene amplification or promoter methylation

**Fig. 1:**
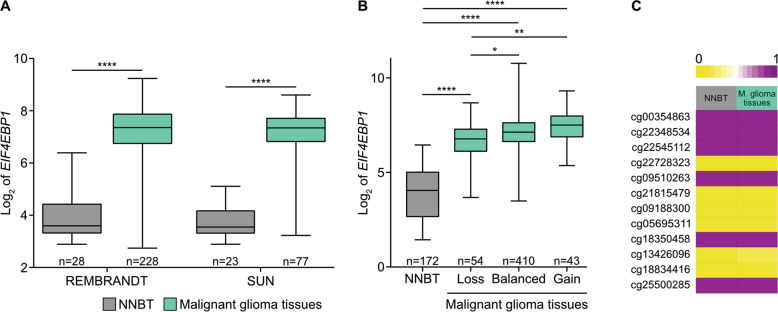
Increased expression of *EIF4EBP1* mRNA in malignant gliomas relative to non-neoplastic brain tissue. **A** Expression levels of *EIF4EBP1* in non-neoplastic brain tissue (NNBT) and glioblastoma tissues from the REMBRANDT [26] and SUN [27] cohorts. **B** Expression levels of *EIF4EBP1* in 172 NNBT samples (BERCHTOLD [67]) and according to *EIF4EBP1* copy number variation in 507 malignant gliomas of CNS WHO grade 4 of TCGA cohort [32] categorized as *EIF4EBP1* copy number loss (hemizygous deletion [loss]), *EIF4EBP1* balanced copy number (balanced), or *EIF4EBP1* low-level copy number gain (gain). **C** DNA methylation levels of 12 CpG sites located within the *EIF4EBP1* promoter region (hg19; Chr8: 37,886,520–37,889,020) using the datasets GSE112179 and GSE156374 for NNBT (*n* = 13) and GSE119774 for malignant glioma (M. glioma) tissues (*n* = 40) with 0 representing unmethylated and 1 representing fully methylated CpG sites. Note identical methylation patterns in normal brain tissue and the glioblastoma samples. Significance in **A**, **B** was calculated using an unpaired and two-tailed parametric *t* test (*p < 0.05, **p < 0.01, ****p < 0.0001).

Based on a glioma dataset from TCGA database, a recent study reported on overexpression of *EIF4EBP1* in glioblastoma tissue samples compared to non-neoplastic brain tissues [12]. To further delineate the expression of *EIF4EBP1* in malignant gliomas including IDH-wildtype and IDH-mutant tumors, we determined the levels of *EIF4EBP1* in additional publically available glioma datasets and investigated its association with common genetic alterations as well as *EIF4EBP1* gene copy number alteration and promoter methylation. We confirmed and extended the reported finding [12] in six independent and non-overlapping patient datasets, namely REMBRANDT [26], SUN [27], FRENCH [28], HEGI [29], TUYSUZ [30], and DONSON [31] (a pediatric glioblastoma dataset). Thereby, we confirmed that malignant glioma tissues showed higher levels of *EIF4EBP1* mRNA expression compared to non-neoplastic brain tissues in each of the analyzed cohorts (Fig. 1A and Fig. S1A, B). We then asked whether *EIF4EBP1* mRNA expression is associated with common genetic and epigenetic alterations found in malignant gliomas. Specifically, we analyzed *EIF4EBP1* mRNA expression levels in *EGFR*-amplified and *EGFR*-non-amplified as well as in O6-methylguanine DNA methyltransferase (*MGMT*) promoter-methylated and promoter-unmethylated IDH-wildtype glioblastoma patient samples using publically available datasets [32]. We found that *EIF4EBP1* mRNA level is not impacted by either of these alterations (Fig. S1C, D). We also investigated the potential association of *EIF4EBP1* expression with the IDH mutation status in primary glioma samples and found that *EIF4EBP1* mRNA expression is not dependent on the IDH mutation status in three independent datasets (Fig. S1E–G). Among IDH-mutant gliomas, there was no difference in *EIF4EBP1* expression levels in 1p/19q-codeleted oligodendrogliomas versus 1p/19q-intact astrocytomas included in the FRENCH cohort dataset [28] (Fig. S1H) or TCGA dataset [32] (Fig. S1I).

Next, we asked whether *EIF4EBP1* overexpression in malignant gliomas might be caused by *EIF4EBP1* gene amplification. Analyzing the copy number status of *EIF4EBP1* in 507 malignant glioma samples did not reveal any amplification of *EIF4EBP1* (Fig. 1B). This observation stands in contrast to a previous report stating that *EIF4EBP1* is amplified in approximately 13% of breast cancers [11]. While approximately 8.5% of TCGA malignant glioma cases analyzed here exhibited a low-level gain of *EIF4EBP1* [33, 34], there was no association with higher *EIF4EBP1* mRNA expression as compared to tumors without *EIF4EBP1* copy number gain (Fig. 1B and Table S1). We then assessed whether *EIF4EBP1* mRNA overexpression is due to differential promoter methylation in non-neoplastic brain versus malignant glioma tissues. We analyzed the DNA methylation level of 12 CpG sites within the *EIF4EBP1* promoter region (hg19; Chr8: 37,886,520–37,889,020), which showed that non-neoplastic brain tissues and malignant glioma tissues exhibited a very similar methylation profile (Fig. 1C). This goes along with a previous study reporting no difference of *EIF4EBP1* promoter methylation in glioma compared to control samples [35]. Based on these analyses, we can exclude *EIF4EBP1* gene amplification or altered *EIF4EBP1* promoter methylation as possible mechanisms driving *EIF4EBP1* overexpression in malignant gliomas.

### Identification of potential transcription factors driving enhanced transcription of *EIF4EBP1* in malignant gliomas

**Fig. 2:**
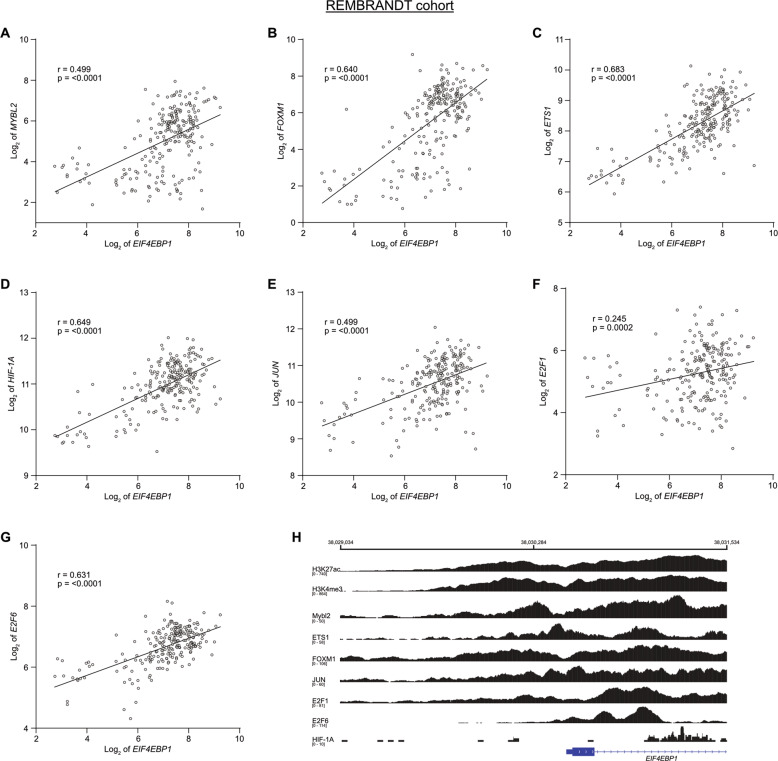
Co-expression of *EIF4EBP1* and *EIF4EBP1* promoter binding transcription factor genes in glioblastoma tissue samples. **A**–**G** Expression levels of *EIF4EBP1* mRNA in glioblastoma patient samples plotted against the mRNA expression levels of (**A**) *MYBL2*, (**B**) *FOXM1*, (**C**) *ETS1*, (**D**) *HIF-1A*, (**E**) *JUN*, (**F**) *E2F1* or (**G**) *E2F6* in the REMBRANDT cohort (*n* = 228 patients) [26]. Co-expression levels were quantified by calculating the Pearson correlation coefficient. **H** ChIP peak locations within the human *EIF4EBP1* promoter, exon 1 and part of intron 1 (−1500 to +1000; hg38; Chr8: 38,029,034–38,031,534) from ChIP-sequencing data for histone H3K27 acetylation (H3K27ac) and H3K4 trimethylation (H3K4me3), ETS1, FOXM1, JUN, E2F1, and E2F6 (Encode consortium, Encyclopedia of DNA Elements at UCSC; [41, 42]), HIF-1A (accession code GSE39089; name GSM955978; run SRR518265 [43]) and MYBL2 (accession code GSE119972; name GSM3389599 [44]).

We next reasoned that the increased *EIF4EBP1* mRNA expression in malignant gliomas might be driven by specific transcription factors. To identify potential transcription factor candidates, we searched for transcription factors that are positively co-expressed with *EIF4EBP1* in malignant gliomas, overexpressed in these tumors as compared to non-neoplastic brain tissues, and known to bind the endogenous *EIF4EBP1* promoter by ChIP. This allowed us to uncover seven transcription factors that fulfilled these criteria. We searched for transcription factors that are positively co-expressed with *EIF4EBP1* in gliomas and found *EIF4EBP1* mRNA expression to be significantly and positively associated with the mRNA expression levels of *MYBL2*, *FOXM1*, *ETS1*, *HIF-1A*, *JUN*, *E2F1*, and *E2F6* in the REMBRANDT dataset [26] (Fig. 2A–G). These associations were validated for each of these transcription factors, excluding *E2F1*, in at least three additional glioma cohorts, including the SUN [27] (Fig. S2A–G), KAWAGUCHI [36], FRENCH [28], or FREIJE [37] datasets (Table S2). In support of the co-expression data, we analyzed the expression of these transcription factors in malignant glioma tissues using TCGA [32, 38] and the REMBRANDT [26] datasets, as well as non-neoplastic brain tissues [39]. This demonstrated a significant overexpression of *MYBL2*, *FOXM1*, *ETS1*, *HIF-1A*, and *JUN* in both glioma cohorts compared to non-neoplastic brain tissues (Fig. S3A, B). Expression of *E2F1* and *E2F6* was previously reported to be higher in glioblastomas (using TCGA dataset) compared to non-neoplastic brain tissues [40], which we validated in the REMBRANDT dataset [26] (Fig. S3B). Of note, the expression of these transcription factors was independent of the IDH mutation status in malignant gliomas, except for *ETS1* (Fig S3C). Finally, we analyzed existing ChIP-sequencing (seq) data from the Encode consortium [41, 42], which demonstrated direct binding of FOXM1, ETS1, E2F1, and E2F6 to the *EIF4EBP1* promoter region, exon 1 and intron 1 (−1500 to +1000) in various normal and cancer cells, however not including glioblastoma cells (Fig. 2H). The transcriptional regulatory region for *EIF4EBP1* is not restricted to its promoter but also encompasses exon 1 and the 5’ region of intron 1, as indicated by histone H3K27 acetylation and H3K4 trimethylation signals (Fig. 2H). In addition, by using other ChIP-seq datasets [43, 44] we found signals for MYBL2 and HIF-1A binding to the *EIF4EBP1* promoter (Fig. 2H). In accordance, ChIP analyses demonstrating HIF-1A binding to its putative responsive element within the *EIF4EBP1* promoter segment −278 to +64 have been published [18]. Taken together, these data indicate that seven transcription factors could contribute to driving increased expression of *EIF4EBP1* in malignant gliomas.

### E2F6, ETS1, HIF-1A, and MYBL2 induce *EIF4EBP1* promoter activity

**Fig. 3:**
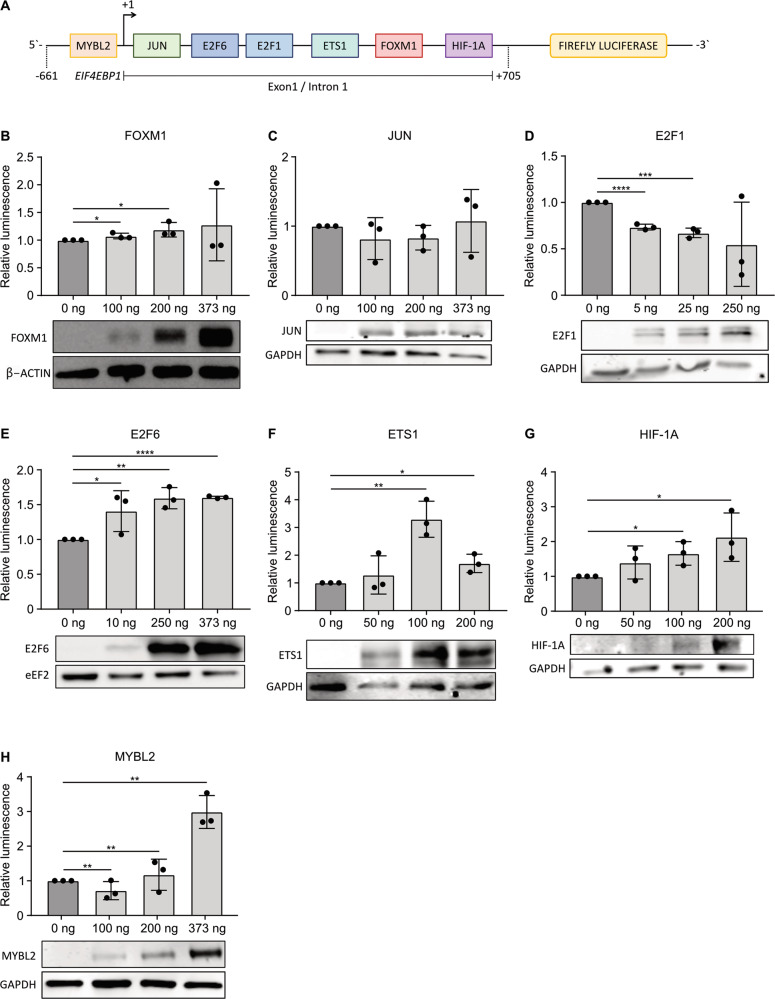
Induction of *EIF4EBP1* promoter activity by E2F6, ETS1, HIF-1A, and MYBL2. **A** Scheme of the luciferase reporter construct containing the *EIF4EBP1* promoter, exon 1, and part of intron 1 (−661; +705), coupled to Firefly luciferase, with the indicated binding sites of transcription factor candidates. **B**–**H** HEK293-T cells were transfected with the −661; +705 *EIF4EBP1* promoter reporter construct, together with increasing amounts of plasmids expressing either one of the indicated transcription factors and a vector expressing *Renilla* luciferase. Luciferase activities were detected using the Dual-Luciferase Reporter Assay. Firefly luciferase activity was normalized to *Renilla* luciferase activity and the ratio was normalized to the corresponding 0 ng condition. Data represent the mean of three independent replicates ± standard deviation (SD). Significance was calculated using an unpaired and one-tailed parametric *t*-test (*p < 0.05, **p < 0.01, ***p < 0.001 ****p < 0.0001). Below each diagram, a representative immunoblot analyzing overexpression of each of the indicated transcription factors is presented.

We next investigated the ability of the seven transcription factor candidates to induce *EIF4EBP1* promoter activity, which was only reported for HIF-1A [18]. To assess promoter activity, we used a luciferase reporter containing the −661 to +705 *EIF4EBP1* promoter region, exon 1, and part of intron 1 (Fig. 3A), as this region is predicted to be bound by the seven transcription factor candidates based on the ChIP-seq data (Fig. 2H). Overexpression of FOXM1 (Fig. 3B) or JUN (Fig. 3C) did not unequivocally induce *EIF4EBP1* promoter activity. While we noticed a significant increase of luciferase activity with low (100 ng) and medium (200 ng) amounts of FOXM1, this was below 1.5-fold and therefore was not considered as biologically relevant. Unexpectedly, overexpression of E2F1, a well-characterized transcriptional activator, led to a decrease of *EIF4EBP1* promoter activity in a dose-dependent manner (Fig. 3D). On the contrary, forced expression of E2F6, a known transcriptional repressor, caused induction of *EIF4EBP1* promoter activity even with low E2F6 expression level (Fig. 3E). Additionally, we showed that ectopic expression of either ETS1 (Fig. 3F), HIF-1A (Fig. 3G), or MYBL2 (Fig. 3H) was able to increase *EIF4EBP1* promoter activity in a dose-dependent manner. The overexpression of each transcription factor was validated by immunoblot analyses (Fig. 3B–H). These experiments proved that among the seven transcription factor candidates, E2F6, ETS1, HIF-1A, and MYBL2 were able to induce *EIF4EBP1* promoter activity. Given that HIF-1A has been previously reported to stimulate *EIF4EBP1* promoter activity [18], we focused on the three other transcription factor candidates for further investigation.

### ETS1 and MYBL2 regulate 4EBP1 mRNA and protein expression

**Fig. 4:**
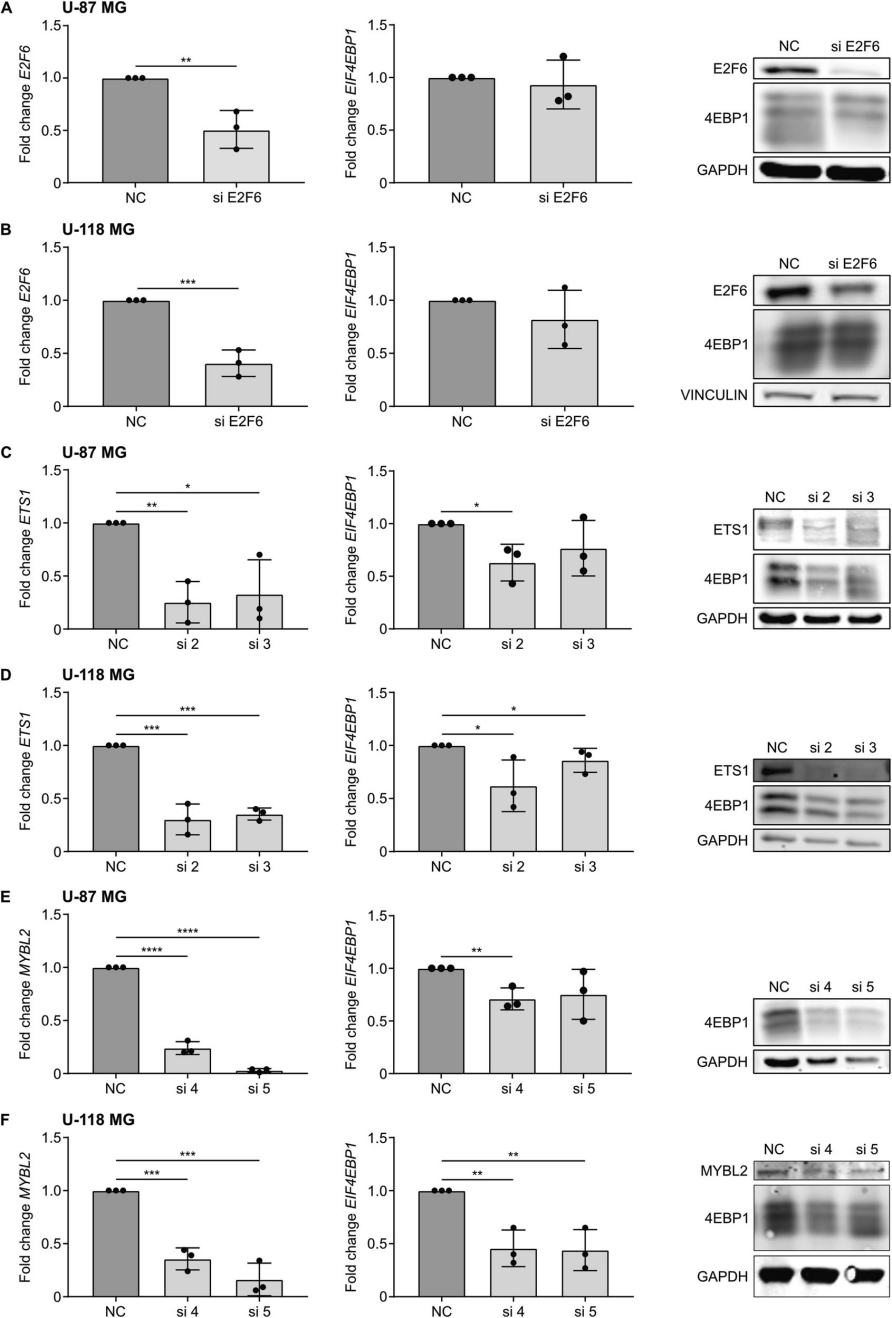
Regulation of *EIF4EBP1* mRNA and protein expression by MYBL2 and ETS1 in glioblastoma cells. **A**–**F** U-118 MG and U-87 MG glioblastoma cells were transiently transfected with negative control siRNAs (NC), and an siRNA pool targeting (**A**, **B**) *E2F6* (si E2F6) or two different siRNAs each targeting either (**C**, **D**) *ETS1* (si 2 and si 3) or (**E**, **F**) *MYBL2* (si 4 and si 5). Cells were re-transfected after 96 h with their corresponding siRNA and incubated for a total of 192 h. MRNA and protein were harvested to determine the expression levels of *EIF4EBP1*/4EBP1 and (**A**, **B**) E2F6, (**C**, **D**) ETS1 or (**E**, **F**) MYBL2 by qRT-PCR and immunoblots. Data obtained by qRT-PCR represent the mean of three independent replicates ±SD and the fold change in expression was normalized to the negative control. Results of representative immunoblot are depicted on the right-hand side of the diagrams representing the qRT-PCR results. Significance was calculated using an unpaired and one-tailed parametric *t*-test (*p < 0.05, **p < 0.01, ***p < 0.001, ****p < 0.0001).

To determine whether ETS1, E2F6, and MYBL2 activate the transcription of endogenous *EIF4EBP1* in glioblastoma cells, each transcription factor was transiently knocked down in U-87 MG and U-118 MG glioblastoma cell lines. At the mRNA level, we achieved at least 50% knock-down for *MYBL2*, *ETS1*, and *E2F6* in both cell lines (Fig. 4A–F). This was confirmed at the protein level, as we observed a decrease of ETS1 and E2F6 in U-118 MG and U-87 MG, and of MYBL2 in U-118 MG upon knock-down. However, while the knock-down of MYBL2 in U-87 MG was strong at the mRNA level, we could not detect it at the protein level due to low endogenous MYBL2 levels in this cell line (Fig. 4E, F). We then assessed the effect of the respective transcription factor knock-downs on 4EBP1 transcript and protein levels. With the half-life of 4EBP1 being longer than 48 h [18], we transfected cells twice with siRNA over a period of 192 h to ensure that 4EBP1 protein is degraded and thus allow for observing potential changes of 4EBP1 protein levels. We observed that E2F6 knock-down in U-87 MG (Fig. 4A) and U-118 MG (Fig. 4B) had no impact on 4EBP1 mRNA and protein levels, eliminating E2F6 as a transcriptional regulator of *EIF4EBP1* in these glioblastoma cell lines. In contrast, transient knock-down of either ETS1 or MYBL2 resulted in a significant decrease of 4EBP1 mRNA and protein levels in both glioblastoma cell lines (Fig. 4C–F). These results were confirmed at the protein levels, i.e., MYBL2 or ETS1 knock-down each resulted in lower 4EBP1 protein levels in both cell lines (Fig. 4C–F). Based on these results, we identified two transcription factors, ETS1 and MYBL2, that regulate *EIF4EBP1* expression in glioblastoma cells.

### *EIF4EBP1* is co-expressed with *MYBL2*, but not with *ETS1*, in other non-CNS cancer types

**Fig. 5:**
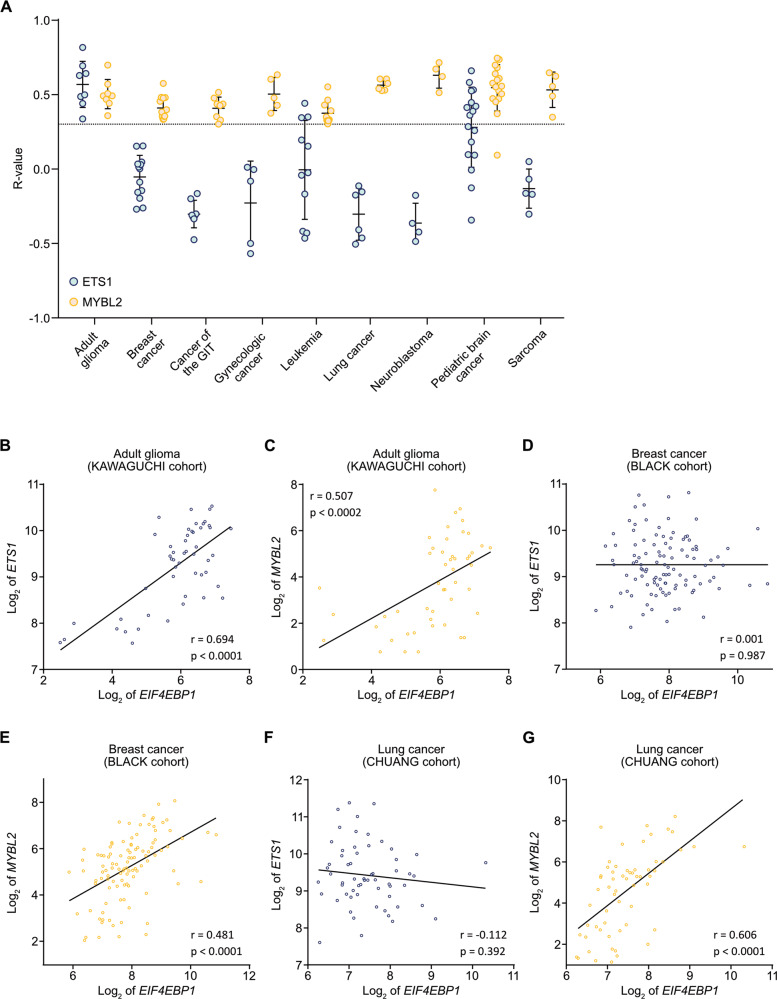
Co-expression of *EIF4EBP1* and *MYBL2* or *ETS1* in different cancer entities. **A** Correlation between the mRNA expression levels of *EIF4EBP1* and *ETS1* (light blue dots) or *MYBL2* (yellow dots) in the indicated human cancer types (Table S3). Co-expression levels were quantified by calculating the Pearson correlation coefficient. Each dot represents the *R*-value for one cohort. The dotted line corresponds to an *R*-value of 0.3, chosen as the cut-off for positive correlation. **B**–**H** Representative co-expression analysis between *EIF4EBP1* mRNA and **B**, **D**, **F** *ETS1* (light blue dots) or **C**, **E**, **G** *MYBL2* (yellow dots) mRNA levels in the indicated tumor type and cohort. The represented cohorts are (**B**, **C**) glioma (KAWAGUCHI cohort; *n* = 50) [36], **D**, **E** breast cancer (BLACK cohort; *n* = 107) [45], and **F**, **G** lung cancer (CHUANG cohort; *n* = 60) cohort [46]. Co-expression levels were quantified by calculating the Pearson correlation coefficient. GIT, gastrointestinal tract.

We further analyzed the potential co-expression of *EIF4EBP1* and either *ETS1* or *MYBL2* at the mRNA level in multiple different cancer types using datasets available in R^2^ AMC (Table S3). These studies indicated that *EIF4EBP1* expression correlates positively with *MYBL2* expression in each of the analyzed tumor entities, whereas co-expression of *EIF4EBP1* with *ETS1* was restricted to CNS tumors (adult-type gliomas and certain pediatric brain cancers) (Fig. 5A). In particular, while we observed that both *MYBL2* and *ETS1* are co-expressed with *EIF4EBP1* in adult-type glioma, as exemplified by the KAWAGUCHI cohort [36] (Fig. 5B, C), only *MYBL2* mRNA levels, but not *ETS1* mRNA levels, showed a positive correlation with *EIF4EBP1* mRNA levels in non-CNS tumor entities, such as breast and lung cancers, as exemplified by the BLACK and CHUANG cohorts [45, 46], respectively (Fig. 5D–G). These analyses indicate that the co-expression between *MYBL2* and *EIF4EBP1* is not restricted to glioblastomas, suggesting that MYBL2 might represent a more general regulatory mechanism driving *EIF4EBP1* expression in different cancer entities.

## Discussion

*EIF4EBP1* gene expression and its clinical relevance in cancer are highly tumor-type specific [47]. We found that *EIF4EBP1* is overexpressed in glioblastoma tissue samples in different patient cohorts as compared to non-neoplastic brain tissues, thus extending previous observations made in the TCGA cohort [12]. Elevated mRNA expression may lead to increased active 4EBP1 protein levels in glioblastoma, as it was reported that mTOR activity is reduced regionally in this tumor entity, thus leading to 4EBP1 activation in poorly vascularized areas [48]. We searched for the underlying causes of increased *EIF4EBP1* mRNA expression in malignant gliomas and observed that the *EIF4EBP1* gene is not amplified in glioblastomas although amplification of 8p11.23, which encompasses *EIF4EBP1*, has been reported in other cancer entities, such as lung squamous cell carcinoma, bladder cancer, and breast cancer, and correlated with higher *EIF4EBP1* expression [49]. By bioinformatic analysis, we identified seven transcription factors that may potentially drive overexpression of *EIF4EBP1* in gliomas. Each of these transcription factors harbors oncogenic or tumor-promoting functions and some of them were reported to be overexpressed in cancer, including overexpression of *E2F1*, *E2F6* [40], *FOXM1*, and *MYBL2* [50] in glioblastomas. Among the seven transcription factor candidates, we found that HIF-1A, E2F6, ETS1, and MYBL2 activated the *EIF4EBP1* promoter in vitro while E2F1, JUN, and FOXM1 did not. Surprisingly, E2F1 a transcriptional activator repressed *EIF4EBP1* promoter activity while E2F6, which is a transcriptional repressor, induced *EIF4EBP1* promoter activity. Of note, E2F1 has been shown to repress transcription of *YAP1* by binding to the transcription factor TEAD [51], so we cannot exclude that E2F1 may repress the endogenous *EIF4EBP1* promoter. While JUN was not validated as a transcriptional regulator of *EIF4EBP1* promoter with our assays, this may be explained by the absence of a consensus binding motif (5’-TGAC/GTCA-3’) [52] within the −661; +705 *EIF4EBP1* promoter construct we used. Of note, the endogenous *EIF4EBP1* promoter contains two JUN consensus binding motifs, which are located further upstream and downstream of the −661; +705 promoter region, suggesting that JUN is still a possible candidate that might regulate the *EIF4EBP1* promoter.

By functional knockdown experiments, we uncovered that ETS1 and MYBL2 regulate the transcription of endogenous *EIF4EBP1* in glioblastoma cells, highlighting novel regulators of *EIF4EBP1* transcription that complement the transcription factors previously reported, including MYC [15], the androgen receptor [16], ATF4 [15], ATF5 [17], and HIF-1A [18]. Since *ETS1* and *MYBL2* as well as *EIF4EBP1* are overexpressed in other cancer entities, for instance in colorectal cancer [12, 13, 53, 54] or breast cancer [12, 53, 55], these transcription factors might also regulate *EIF4EBP1* expression in cancers outside the CNS. In support of this assumption, we found that *MYBL2*, but not *ETS1*, is co-expressed with *EIF4EBP1* at the mRNA level in a variety of non-CNS cancer entities, suggesting that MYBL2 might represent a general transcriptional driver of *EIF4EBP1* overexpression in human cancers while ETS1-dependent regulation of *EIF4EBP1* may be more restricted to CNS tumors. The molecular mechanisms underlying *MYBL2* and *ETS1* overexpression in malignant gliomas are to date unknown. In the case of *MYBL2*, this may be due to EGFR signaling, which is frequently amplified and overexpressed in IDH-wildtype glioblastomas [56] and was reported to activate the *MYBL2* promoter in association with E2F1 [57]. ETS1 activity is directly induced by the RAS/RAF/MEK/ERK pathway [53], which is overactive in a large number of IDH-wildtype glioblastomas [58] and leads to *ETS1* promoter activation [53].

Given that we found *EIF4EBP1* to be a target gene of the ETS1 and MYBL2 oncoproteins in malignant gliomas, 4EBP1 may possibly contribute to ETS1 and MYBL2 tumorigenic functions in these tumors. Functions of both transcription factors as well as 4EBP1 have been linked to support angiogenesis. Indeed, ETS1 is known to regulate the *VEGF* promoter and its transcription [59], and ETS1 expression is associated with a higher density of microvessels in tumors [60]. MYBL2 expression was reported to be induced under ischemic conditions in rat brains [61], stabilized by HIF-2α [62], and to protect cells toward hypoxia-induced apoptosis [63]. Additionally, 4EBP1 has been shown to promote the selective translation of *VEGF* or *HIF-1A* mRNAs in response to hypoxia [7]. Taken together, this raises the possibility that the induction of *EIF4EBP1* expression by ETS1 and MYBL2 in glioblastoma cells may be a previously unrecognized mechanism mediating angiogenesis in this tumor type. Independently of ETS1 or MYBL2, 4EBP1 may exhibit other functions in glioblastomas. It has been reported that 4EBP1 is required for oncogenic RAS transformation of mouse embryonic fibroblasts in vitro and in vivo [64], pointing to a tumor-supporting role of 4EBP1. Thus, it is possible that 4EBP1 may also contribute to glioma tumorigenesis by supporting oncogenicity.

In summary, we elucidated molecular mechanisms of enhanced *EIF4EBP1* levels in glioblastoma cells, revealing the oncogenic transcription factors ETS1 and MYBL2 as responsible transcriptional regulators.

## Materials and methods

### Data availability and bioinformatics analysis

We used publically available cancer datasets (Table S3) as well as glioma and non-neoplastic brain tissue datasets derived from various cohorts for correlative analyses of RNA expression data. Table S4 provides an overview of the glioma datasets that were used including accession numbers, patient numbers, original diagnoses, and information on IDH mutation status, if available. As these datasets were generated before the current WHO classification, the provided diagnoses are mostly based on histological classification only. RNA expression data were analyzed with the Gepia website [38] using the publicly available GTEx non-neoplastic brain tissue and TCGA [32] (tumor tissues) datasets or obtained from the R^2^ Genomic Analysis Visualization Platform (R^2^ AMC; http://r2.amc.nl) using the REMBRANDT [26] datasets to analyze the expression levels of *EIF4EBP1*, *MYBL2*, *FOXM1*, *ETS1*, *HIF-1A*, *JUN*, *E2F1*, and *E2F6* in non-neoplastic brain tissue versus malignant glioma patient samples. Additionally, the expression levels of *EIF4EBP1* were analyzed with R^2^ AMC using the SUN [27], FRENCH [28], HEGI [29], DONSON [31] (microarray platforms u133p2) and TUYSUZ [30] (microarray platform hugene21t) datasets. For co-expression analyses, the above-mentioned cohorts as well as the KAWAGUCHI [36], FREIJE [37], and PAUGH [65] cohorts were used. Expression data of IDH-wildtype glioblastoma patient samples according to the *MGMT* promoter methylation status were retrieved from cBioportal [33, 34] (TCGA [32]) and data related to the *EGFR* amplification status in IDH-wildtype glioblastomas were retrieved with R^2^ AMC using the FRENCH [28] cohort. Expression data according to 1p/19q codeletion were obtained for IDH-mutant CNS WHO grade 2, 3, and 4 gliomas from R^2^ AMC using the FRENCH [28] cohort or from https://portal.gdc.cancer.gov using TCGA datasets for lower-grade glioma and glioblastoma [32]. MRNA expression data according to IDH mutation status were analyzed using the CGGA [66], FRENCH [28], and TCGA [32] datasets for *EIF4EBP1* expression and TCGA dataset [32] for the expression of the transcription factors. TCGA data were accessed using cBioportal [33, 34]. Copy number variations for *EIF4EBP1* and corresponding *EIF4EBP1* expression in glioma patient samples were acquired from cBioportal and R^2^ AMC, respectively [33, 34] (TCGA [32]) and compared to expression data of *EIF4EBP1* in non-neoplastic brain tissue [67] from R^2^ AMC. DNA methylation data were downloaded from R^2^ AMC (GSE112179 [68] and GSE156374 [69] for non-neoplastic brain tissue and GSE119774 [70] for tumor tissues). CpG sites included within the −1500 to +1000 of *EIF4EBP1* (human genome GRCh 38/hg38; Chr8: 38,029,034–38,031,534) were selected for analysis and the mean was determined for each group and CpG site. ChIP-seq data for H3K27ac (UCSC Accession: wgEncodeEH000030, wgEncodeEH000997, wgEncodeEH000111, wgEncodeEH000055, wgEncodeEH000043, wgEncodeEH000064, wgEncodeEH000097), H3K4me3 (wgEncodeEH000913, wgEncodeEH000909, wgEncodeEH002876, wgEncodeEH001882), ETS1 (wgEncodeEH002290; wgEncodeEH001580), FOXM1 (wgEncodeEH002529), JUN (wgEncodeEH000746, wgEncodeEH000719, wgEncodeEH002805, wgEncodeEH000620), E2F1 (wgEncodeEH000699, wgEncodeEH000688, wgEncodeEH000693) and E2F6 (wgEncodeEH000692 wgEncodeEH000676; wgEncodeEH001598) were downloaded from ENCODE (Encyclopedia of DNA Elements at UCSC; [41, 42]) using the human genome GRCh 38/hg 38, whereas NCBI Geo datasets were used to access ChIP-seq data for HIF-1A (human genome GRCh 38/hg 38; accession code GSE39089; name GSM955978; run SRR518265; [43]) and MYBL2 (human genome GRCh 37/hg 19; accession code GSE119972; name GSM3389599; [44]). Fastq files for HIF-1A were aligned to human reference genome hg38 using STAR v2.4.1d, whereas MYBL2 data were re-aligned from hg19 to hg38. ChIP seq data from ENCODE [41, 42] included data from seven cell lines. These files were combined into a single BAM file. BAM files were then visualized using IGV version 2.9.1 (https://igv.org; [71]).

### Statistical analyses

Unpaired *t*-tests were performed when comparing gene expression in gliomas versus non-neoplastic brain tissues samples, as well as between IDH-mutant glioma groups stratified according to 1p/19q co-deletion, or IDH-wildtype glioblastoma groups stratified according to *EGFR* amplification and *MGMT* promoter methylation status. ANOVA analysis was used to determine the significance of copy number status between glioma and non-neoplastic brain tissue samples. Correlation analyses were performed by calculating Pearson correlation. GraphPad Prism version 7.04 (GraphPad Software, San Diego, CA, USA) was used for the statistical analysis.

### Cell culture

HEK293-T embryonic kidney cells as well as the human glioblastoma cell lines U-118 MG and U-87 MG were originally obtained from American Type Culture Collections (ATCC). Cells were maintained in Dulbecco’s modified Eagle Medium (10569010, Thermo Fisher Scientific, Waltham, MA, USA) supplemented with 10% fetal bovine serum (FBS) (10270-106, Thermo Fisher Scientific) and 1% penicillin/streptomycin (10270-106, Sigma Aldrich, St Louis, USA) and cultured in a humidified incubator at 37 °C with 5% CO_2_. The cell lines were confirmed to be mycoplasma-free by Venor GeM Classic (11-1050, Minerva Biolabs, Berlin, Germany) kit and validated by STR-profiling (Genomics & Transcriptomics Labor (GTL), Heinrich Heine University, Düsseldorf, Germany).

### siRNA transfection

Cells were transfected in 6-well plates at 70% confluency with 25 nM control siRNA (D-001206-14-50, Dharmacon, Cambridge, UK) or negative control siPool (siTools Biotech, Planegg, Germany) or siRNAs targeting *ETS1* (D-003887-02-0010 & D-003887-03-0010, Dharmacon), *MYBL2* (D-010444-04-0005 and D-010444-05-0005, Dharmacon) or *E2F6* (siTools Biotech) using siLentFect transfection reagent (1703362, Biorad, Hercules, CA, USA) (see Table S5 for siRNA sequences). Briefly, a master mix containing 125 µl Opti-MEM (31985-070, Thermo Fisher Scientific) and 3 µl siLentFect was prepared and incubated for 5 min at room temperature (RT). Meanwhile, 125 µl Opti-MEM were mixed with 25 nM of siRNA for each well. The siRNA mix was mixed 1:1 with the master mix, incubated for 20 min at RT, and added dropwise onto the cells. The medium was changed the day after transfection. Cells were re-transfected after 96 h. At 192 h following the first transfection, RNA and protein were harvested for further analysis.

### Plasmid construction

The promoter region of the human *EIF4EBP1* gene, spanning from nucleotide −661 to +705 (human genome GRCh 38/hg38; Chr8: 38,029,873–38,031,239), was inserted into the SacI and BglII restriction sites of the Firefly Luciferase expressing pGL4.22 plasmid (E6771, Promega, Madison, WI, USA). Cloning was performed by GENEWIZ Germany GmbH (Leipzig, Germany).

### Luciferase reporter assays

HEK 293-T cells were seeded in 12-well plates to reach 50% confluency on the day of transfection. Cells were transfected with 125 ng of the *EIF4EBP1* promoter Firefly luciferase plasmid, 2 ng of *Renilla* luciferase-expressing pRL SV40 plasmid (E2231, Promega), as internal control, and 5–373 ng of either of the transcription factor expressing plasmids, completed to 500 ng total DNA with pCMV-Neo-Bam (16440, Addgene) or pcDNA3.1 (V79020, Thermo Fisher Scientific) plasmids using CalFectin^TM^ Cell Transfection Reagent (SL100478, SignaGen Laboratories; Frederick, MD; USA) according to the manufacturer’s guidelines. The used transcription factor expressing plasmids were pcDNA3 E2F1 (kind gift from Dr. Tony Kouzarides, University of Cambridge, UK), pSG3.1 ETS1 (kindly provided by Dr. Lawrence McIntosh, University of British Columbia, Vancouver, Canada), pcDNA3 FoxM1 (kindly provided by Dr. Pradip Raychaudhuri, University of Illinois Cancer Center, Chicago, IL, USA), pcDNA3 HA-HIF-1A (gift from Dr. William Kaelin [Addgene plasmid # 18949; http://n2t.net/addgene:18949; RRID:Addgene_18949; [72]]), pcDNA3 MYBL2 (gift from Dr. Rob Lewis [Addgene plasmid # 25965; http://n2t.net/addgene:25965; RRID:Addgene_25965; [73]]), pCMV6 JUN (kind gift of Dr. Marguerite Buzza, University of Maryland, College Park, MD, USA). Cells were harvested 48 h post-transfection and the activity of Firefly and *Renilla* luciferases were sequentially determined using the Dual-Luciferase Reporter Assay System (E1980, Promega) and analyzed with Beckman Coulter microtiter plate reader (Beckman Coulter, Krefeld, Germany). All samples were performed in triplicate and the final luciferase quantification was formulated as the ratio of Firefly luciferase to *Renilla* luciferase luminescence.

### RNA extraction, cDNA synthesis, and qRT-PCR

RNA was extracted using the RNeasy Plus Mini Kit (74136, QIAgen, Hilden, Germany). The extraction was performed according to the protocol provided by the manufacturer. Isolated RNA was retro-transcribed to cDNA using 1 μg of RNA per reaction with either the QuantiTect Reverse Transcription Kit (205311, QIAgen) or the High-Capacity cDNA Reverse Transcription Kit (4368813, Applied Biosystems, Waltham, MA, USA) according to the manufacturer’s protocol. Real-time PCR was performed in triplicates using 1 µl cDNA and 9 µl master mix consisting of 5 µl SYBR Green PCR Mix (4309155, Applied Biosystems), 3 µl H_2_O and 1 µl of forward and reverse primers (0.5 µM final concentration). PPIA, GusB, and β-actin were used as housekeepers. For primer sequences, see Table S6.

### Protein extraction and immunoblot analysis

Cells were lysed in RIPA buffer (150 mM NaCl, 50 mM Tris-HCl, pH 8, 1% Triton X100, 0.5% Sodium deoxycholate, and 0.1% SDS) supplemented with proteinase inhibitor cocktail (11873580001, Roche, Basel, Switzerland) and phosphatase inhibitor (04906837001, Roche). Cell lysates were centrifuged at 14,000 × *g* for 15 min at 4 °C and supernatants were collected. Protein concentration was quantified using the Pierce^TM^ BCA Protein Assay Kit (23225, Thermo Fisher Scientific) according to the manufacturer’s protocol. Twenty micrograms of total protein were loaded either on a 12% polyacrylamide-SDS gel or on a NativePAGE™ 4–12%, Bis-Tris Gels (NP0336BOX, Thermo Fisher Scientific) and transferred to a 0.2 µm nitrocellulose membrane (No10600001, GE Healthcare; Chicago, IL, USA). Membranes were blocked with 5% bovine serum albumin (BSA) (8076.3, Carl Roth, Karlsruhe, Germany) TBS-Tween (20 mM Tris-HCl, pH 7.4, 150 mM NaCl, 0.1% Tween 20) and probed with primary antibodies (as detailed in table S7) diluted 1:1000 in 5% BSA TBS overnight at 4 °C if not stated otherwise. Membranes were then incubated with a corresponding anti-mouse (926-32210, Li-Cor, Bad Homburg, Germany) or anti-rabbit (926-32211, Li-Cor) fluorescent secondary antibody diluted 1:10,000. The fluorescent signal was visualized with the LI-COR Odyssey® CLx system (Li-Cor).

### Statistical analysis of experimental data

All experiments were carried out in three biological replicates. Data are represented as mean +/− standard deviation (SD). A one- or two-sided Student’s *t*-test was used to compare differences between control and experimental groups. Results were considered as being statistically significant at *p* < 0.05. Statistical tests were calculated with GraphPad Prism version 7.04.

## Supplementary information

Original Data File

Original Data File

Supplementary legends

Supplementary Figure 1

Supplementary Figure 2

Supplementary Figure 3

Supplementary Tables

## Data Availability

The data that support the findings of this study are available from the corresponding author upon reasonable request.
