# Supplementary figures and images for: Eukaryotic translation initiation factor 4E binding protein 1 (EIF4EBP1) expression in glioblastoma is driven by ETS1- and MYBL2-dependent transcriptional activation
Source: Cell Death Discov. 2022 Feb 28;8:91. doi: 10.1038/s41420-022-00883-z (PMC8885828; doi:10.1038/s41420-022-00883-z)

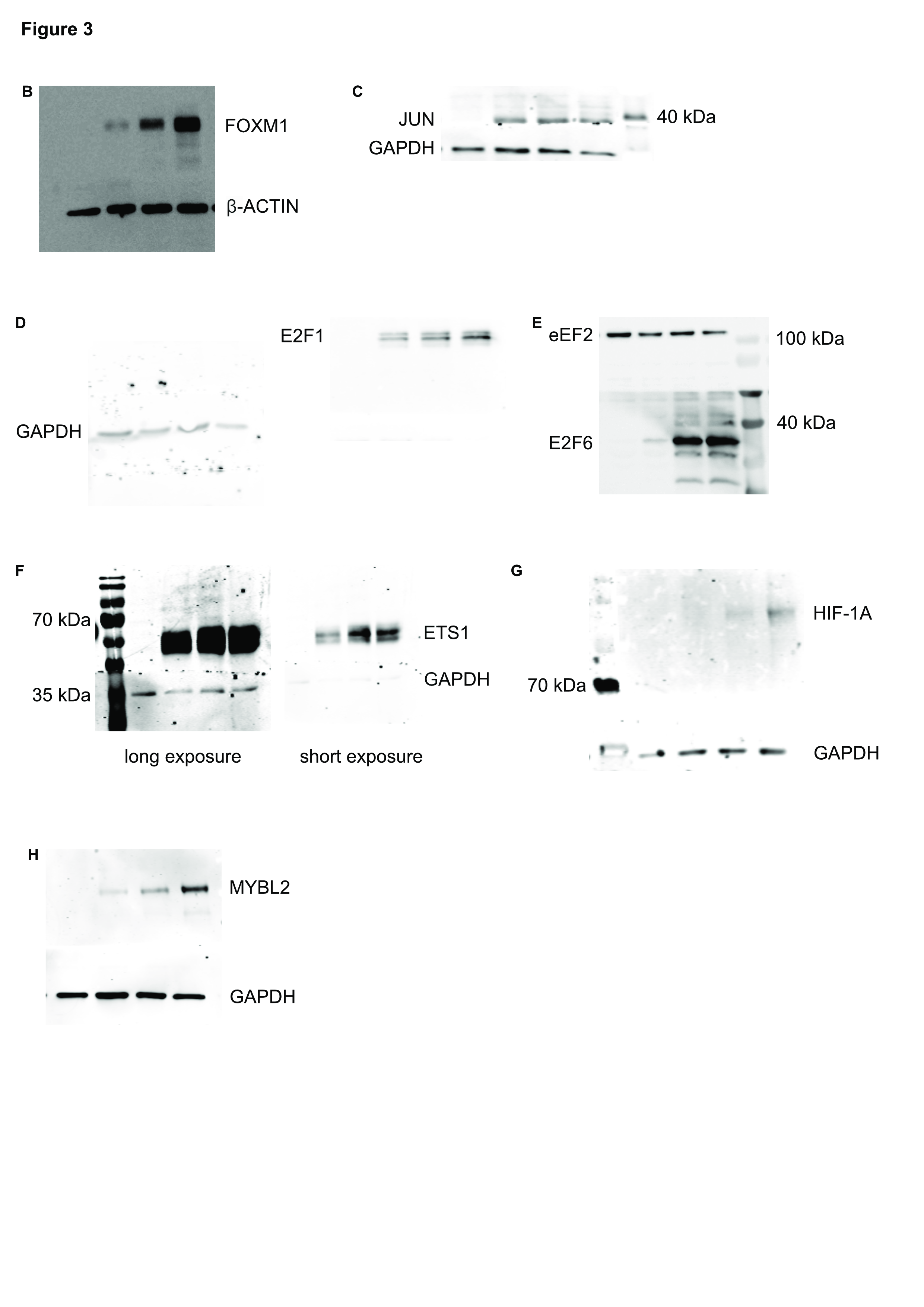

Supplement: Supplementary file 1 — Original Data File [file 41420_2022_883_MOESM1_ESM.tif]

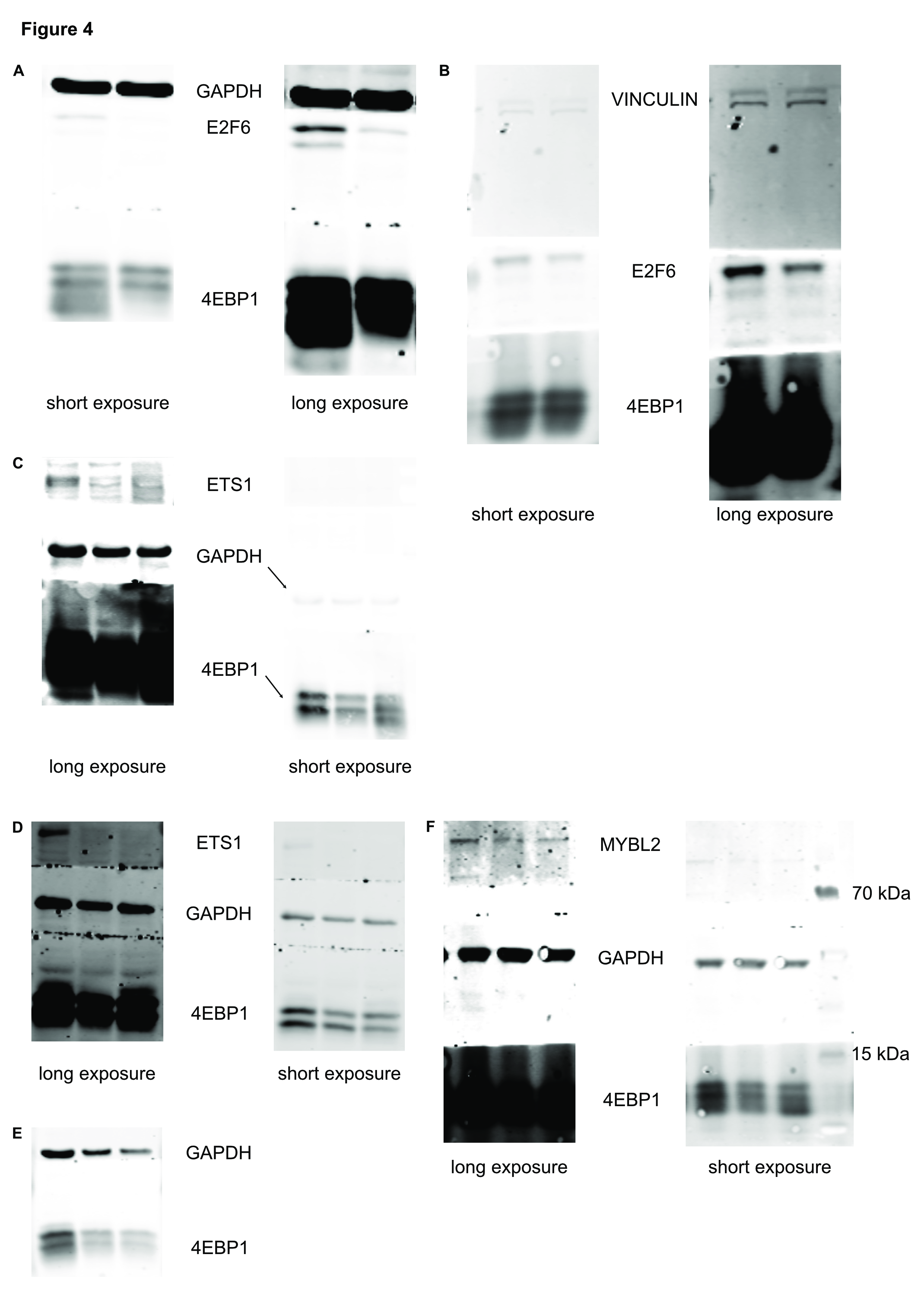

Supplement: Supplementary file 2 — Original Data File [file 41420_2022_883_MOESM2_ESM.tif]

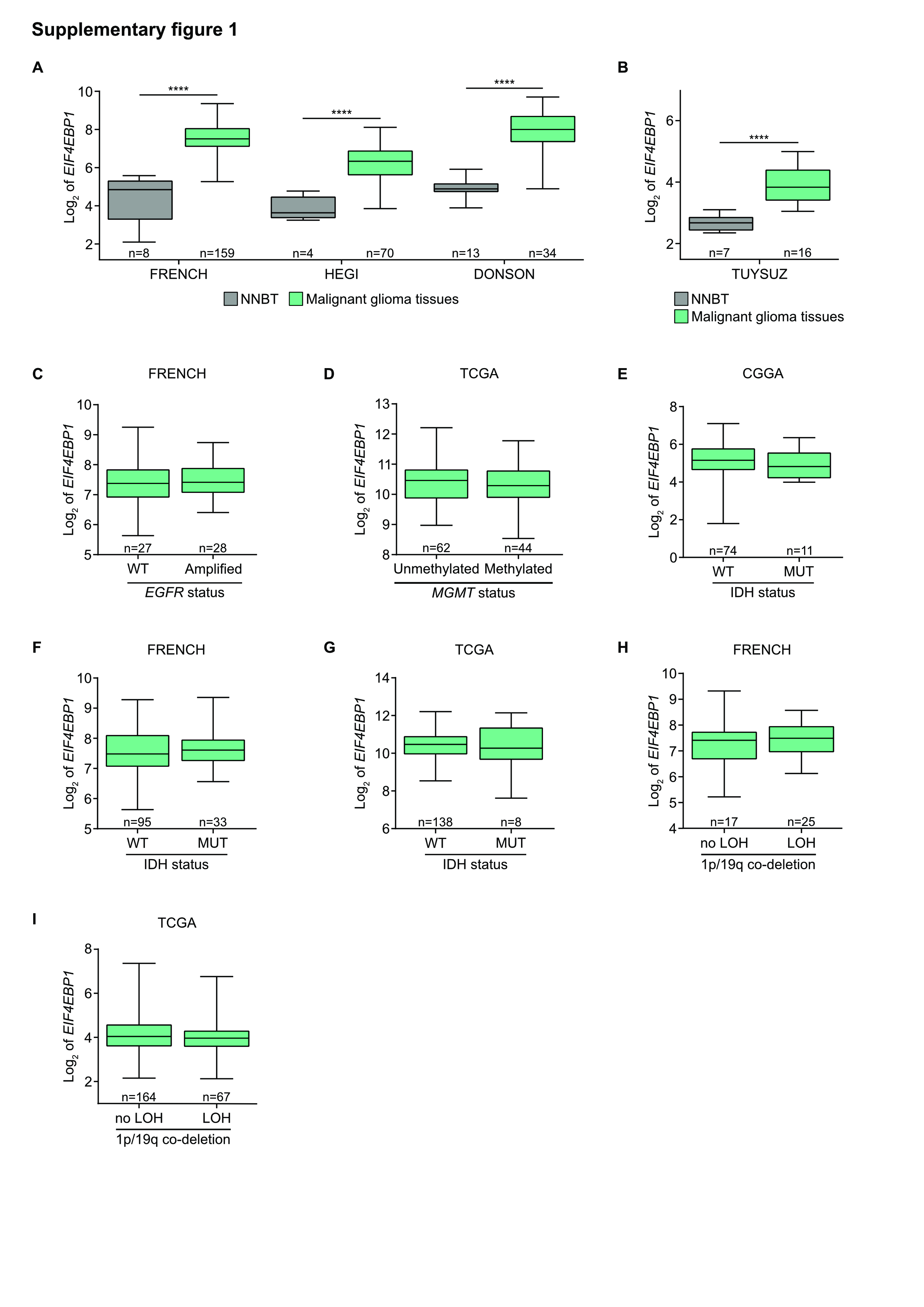

Supplement: Supplementary file 4 — Supplementary Figure 1 [file 41420_2022_883_MOESM4_ESM.tif]

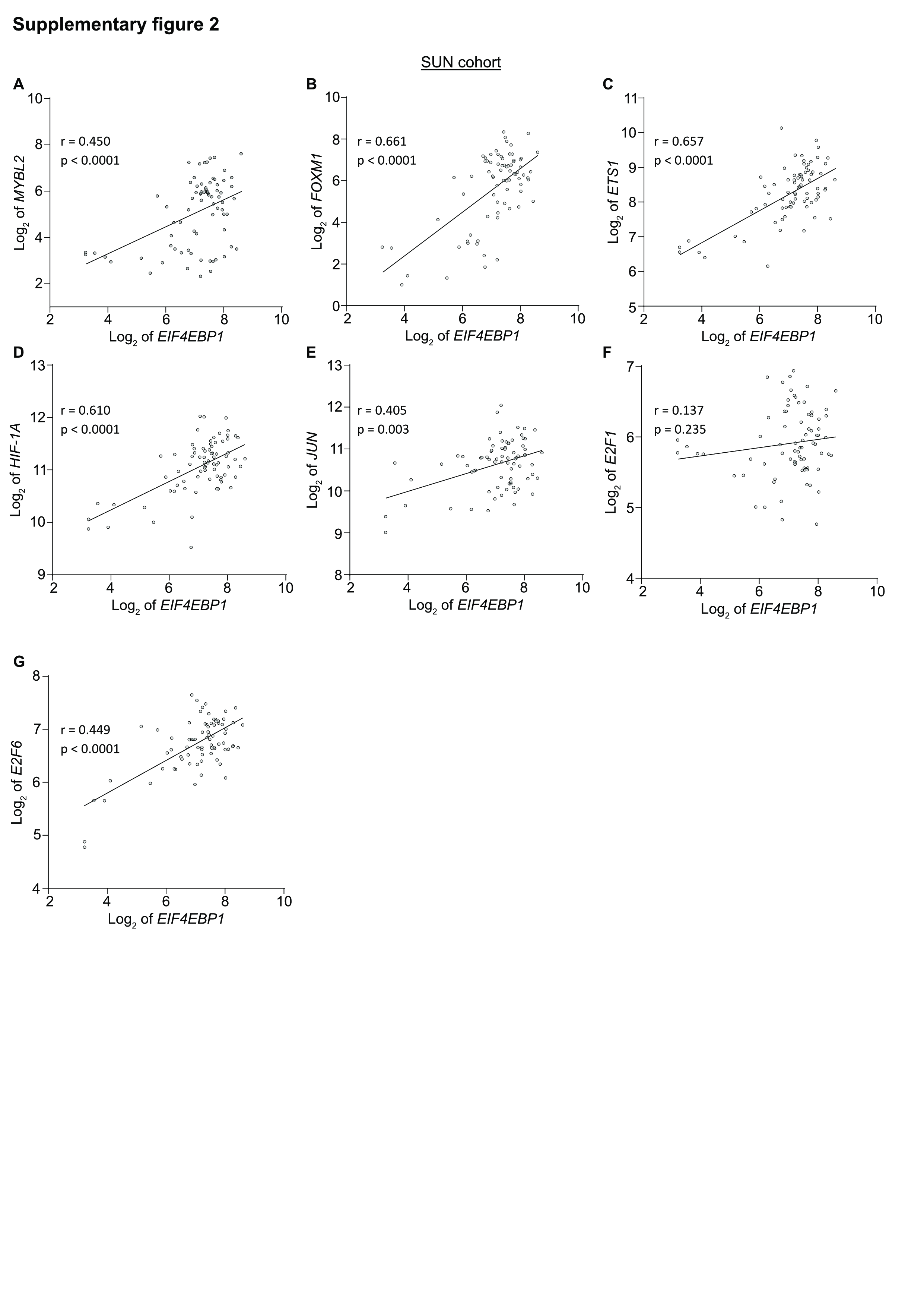

Supplement: Supplementary file 5 — Supplementary Figure 2 [file 41420_2022_883_MOESM5_ESM.tif]

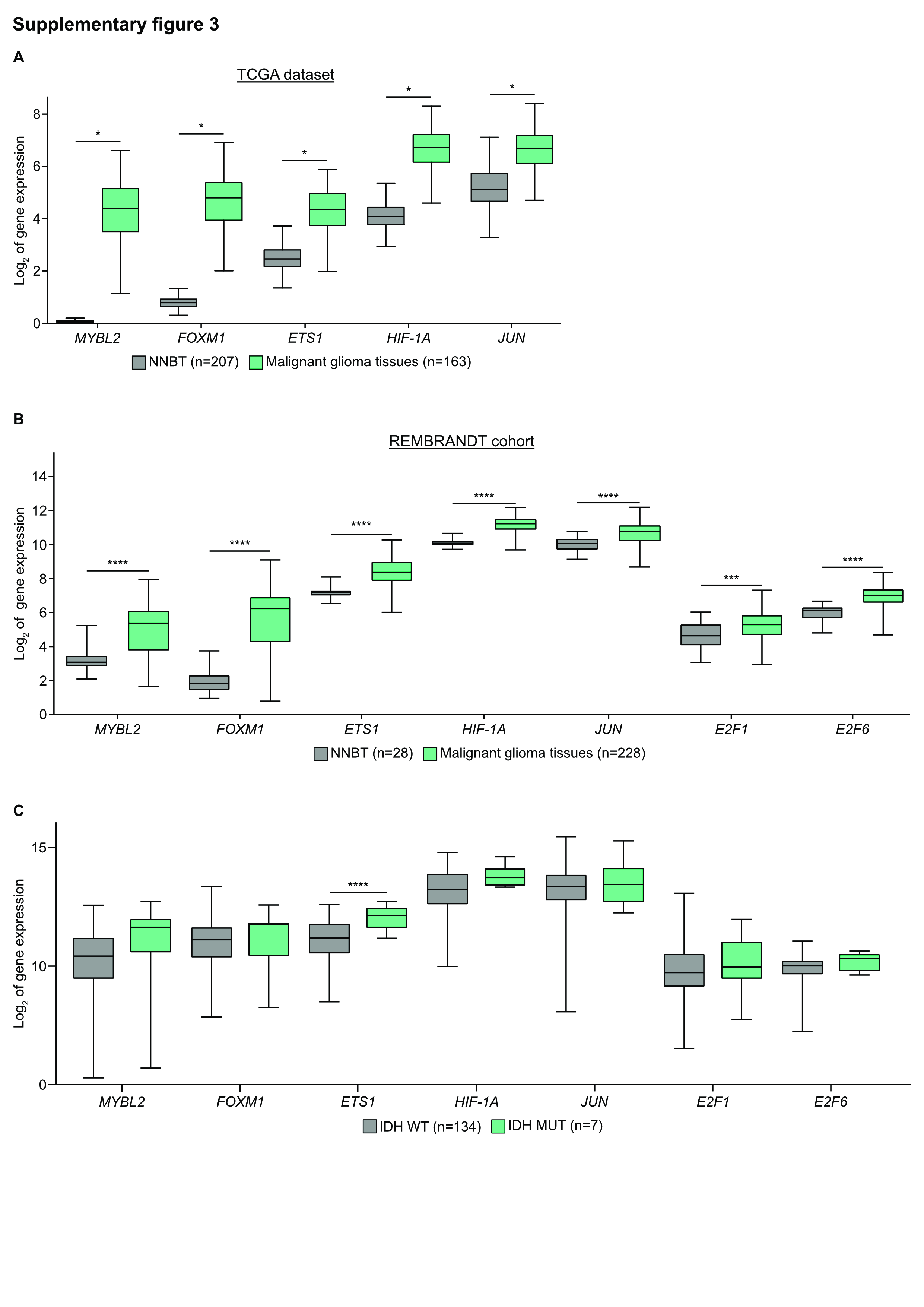

Supplement: Supplementary file 6 — Supplementary Figure 3 [file 41420_2022_883_MOESM6_ESM.tif]
