# Supplementary material for: Eukaryotic translation initiation factor 4E binding protein 1 (EIF4EBP1) expression in glioblastoma is driven by ETS1- and MYBL2-dependent transcriptional activation
Source: Cell Death Discov. 2022 Feb 28;8:91. doi: 10.1038/s41420-022-00883-z (PMC8885828; doi:10.1038/s41420-022-00883-z)
Supplement: Supplementary file 3 — Supplementary legends [file 41420_2022_883_MOESM3_ESM.docx]

**SUPPLEMENTARY FIGURE LEGENDS**

**Supplementary Figure 1. *EIF4EBP1* expression malignant gliomas in relation to non-neoplastic brain tissue and according to selected genetic and epigenetic alterations.**

A and B, Expression levels of *EIF4EBP1* in non-neoplastic brain tissue (NNBT) and in glioblastoma/malignant glioma tissue samples from the indicated patient cohorts, i.e. FRENCH (1), HEGI (2), DONSON (3) (microarray platforms u133p2), and TUYSUZ (4) (microarray platform hugene21t). C, Expression levels of *EIF4EBP1* in *EGFR*-non-amplified (WT) versus *EGFR*-amplified IDH-wildtype glioblastoma tissues of the FRENCH cohort (1). D, Expression levels of *EIF4EBP1* in IDH-wildtype glioblastoma samples of TCGA cohort (5) according to *MGMT* promoter methylation status. E-G, Expression levels of *EIF4EBP1* according to IDH mutation status in CNS WHO grade 4 glioma samples of the (E) CGGA (6), (F) FRENCH (1) and (G) TCGA cohorts (5). H and I, Expression levels of *EIF4EBP1* in CNS WHO grade 2-4 IDH-mutant diffuse glioma samples of the (H) FRENCH (1) and (I) TCGA (5) cohorts stratified according to 1p/19q codeletion status. Significance was calculated using unpaired and two-tailed parametric t-tests (****p<0.0001).

**Supplementary Figure 2. Co-expression of *EIF4EBP1* with seven transcription factor genes in glioblastomas.**

A-G, Expression levels of *EIF4EBP1* in glioblastoma patient samples plotted against the expression levels of (A) *MYBL2*, (B) *FOXM1*, (C) *ETS1*, (D) *HIF-1A*, (E) *JUN*, (F) *E2F1* or (G) *E2F6* in the SUN cohort of 77 glioblastoma samples (7). Co-expression levels were quantified by calculating the Pearson correlation coefficient.

**Supplementary Figure 3. Elevated expression of transcription factor candidate genes in malignant gliomas.**

A and B, Expression levels of the indicated transcription factor genes in non-neoplastic brain tissue (NNBT) of GTEx and in malignant glioma tissues from (A) TCGA (5), which analysed subcohort contains 138 IDH-wildtype, 8 IDH-mutant and 17 malignant glioma samples with unknown IDH status, or (B) REMBRANDT (8) cohorts. Significance for the REMBRANDT data was calculated using unpaired and two-tailed parametric t-tests (***p<0.001, ****p<0.0001). Significance for TCGA data has been calculated by GEPIA website using one-way ANOVA (9). C, Expression levels of the indicated transcription factor genes according to IDH mutation status in malignant glioma tissues were analysed using TCGA data (5). Significance was calculated using unpaired and two-tailed parametric t-tests (****p<0.0001).

**REFERENCES**

1. Gravendeel LA, Kouwenhoven MC, Gevaert O, de Rooi JJ, Stubbs AP, Duijm JE, et al. Intrinsic gene expression profiles of gliomas are a better predictor of survival than histology. Cancer research. 2009;69(23):9065-72.

2. Murat A, Migliavacca E, Gorlia T, Lambiv WL, Shay T, Hamou MF, et al. Stem cell-related "self-renewal" signature and high epidermal growth factor receptor expression associated with resistance to concomitant chemoradiotherapy in glioblastoma. J Clin Oncol. 2008;26(18):3015-24.

3. Griesinger AM, Birks DK, Donson AM, Amani V, Hoffman LM, Waziri A, et al. Characterization of distinct immunophenotypes across pediatric brain tumor types. Journal of immunology. 2013;191(9):4880-8.

4. Gulluoglu S, Tuysuz EC, Sahin M, Kuskucu A, Kaan Yaltirik C, Ture U, et al. Simultaneous miRNA and mRNA transcriptome profiling of glioblastoma samples reveals a novel set of OncomiR candidates and their target genes. Brain Res. 2018;1700:199-210.

5. Cancer Genome Atlas Research N, Weinstein JN, Collisson EA, Mills GB, Shaw KR, Ozenberger BA, et al. The Cancer Genome Atlas Pan-Cancer analysis project. Nat Genet. 2013;45(10):1113-20.

6. Zhao Z, Zhang KN, Wang Q, Li G, Zeng F, Zhang Y, et al. Chinese Glioma Genome Atlas (CGGA): A Comprehensive Resource with Functional Genomic Data from Chinese Glioma Patients. Genomics Proteomics Bioinformatics. 2021;19(1):1-12.

7. Sun L, Hui AM, Su Q, Vortmeyer A, Kotliarov Y, Pastorino S, et al. Neuronal and glioma-derived stem cell factor induces angiogenesis within the brain. Cancer Cell. 2006;9(4):287-300.

8. Gusev Y, Bhuvaneshwar K, Song L, Zenklusen JC, Fine H, Madhavan S. The REMBRANDT study, a large collection of genomic data from brain cancer patients. Sci Data. 2018;5:180158.

9. Tang Z, Li C, Kang B, Gao G, Li C, Zhang Z. GEPIA: a web server for cancer and normal gene expression profiling and interactive analyses. Nucleic Acids Res. 2017;45(W1):W98-W102.
