# Supplementary material for: Eukaryotic translation initiation factor 4E binding protein 1 (EIF4EBP1) expression in glioblastoma is driven by ETS1- and MYBL2-dependent transcriptional activation
Source: Cell Death Discov. 2022 Feb 28;8:91. doi: 10.1038/s41420-022-00883-z (PMC8885828; doi:10.1038/s41420-022-00883-z)
Supplement: Supplementary file 7 — Supplementary Tables [file 41420_2022_883_MOESM7_ESM.docx]

**Supplementary table 1: Copy number status of *EIF4EBP1* in malignant gliomas of the TCGA cohort.**

| **Copy number status** | **Number of cases (% of entire cohort)** |
| --- | --- |
| Copy number loss (hemizygous deletion) | 54 (10,63 %) |
| Balanced copy number | 410 (80,71 %) |
| Low-level copy number gain | 43 (8,46 %) |
| High level copy number gain (amplification) | 0 (0 %) |

**Supplementary table 2: Overview of the co-expression analysis between *EIF4EBP1* and the different transcription factor gene candidates in the various glioma cohorts analyzed.**

| **Transcription factor** | **Cohort** | **r-value** | **p-value** | **Total number of cohorts** |
| --- | --- | --- | --- | --- |
| **MYBL2** | SUN (1) | 0.450 | 4.13-05 | 8 |
|  | REMBRANDT (2, 3) | 0.499 | 9.2e-16 |  |
|  | DONSON (4) | 0.587 | 2.6e-04 |  |
|  | HEGI (5) | 0.367 | 1.8e-03 |  |
|  | TCGA (6) | 0.403 | 3.2e-07 |  |
|  | FREIJE (7) | 0.393 | 7.1e-04 |  |
|  | FRENCH (8) | 0.321 | 3.7e-05 |  |
|  | KAWAGUCHI (9) | 0.465 | 7.3e-03 |  |
|  | | | | |
| **FOXM1** | SUN (1) | 0.661 | 6.1e-11 | 8 |
|  | REMBRANDT (2, 3) | 0.640 | 1.1e-27 |  |
|  | DONSON (4) | 0.584 | 2.9e-04 |  |
|  | HEGI (5) | 0.383 | 1.1e-03 |  |
|  | TCGA (6) | 0.338 | 3.5e-11 |  |
|  | FREIJE (7) | 0.597 | 4.0e-08 |  |
|  | KAWAGUCHI (9) | 0.662 | 3.7e-05 |  |
|  | PAUGH (10) | 0.445 | 9.4e-03 |  |
|  | | | | |
| **ETS1** | SUN (1) | 0.657 | 8.8e-11 | 6 |
|  | REMBRANDT (2, 3) | 0.683 | 1.2e-32 |  |
|  | DONSON (4) | 0.509 | 2.1e-03 |  |
|  | HEGI (5) | 0.468 | 4.5e-05 |  |
|  | FRENCH (8) | 0.241 | 2.3e-03 |  |
|  | KAWAGUCHI (9) | 0.593 | 0.5e-04 |  |
|  | | | | |
| **HIF-1A** | SUN (1) | 0.610 | 3.97e-09 | 4 |
|  | REMBRANDT (2, 3) | 0.596 | 4.41e-54 |  |
|  | FREIJE (7) | 0.528 | 2.22e-06 |  |
|  | KAWAGUCHI (9) | 0.637 | 8.89e-05 |  |
|  | | | | |
| **JUN** | SUN (1) | 0.405 | 2.6e-04 | 7 |
|  | TUYSUZ (11) | 0.565 | 0.02 |  |
|  | REMBRANDT (2, 3) | 0.499 | 9.7e-16 |  |
|  | HEGI (5) | 0.378 | 1.2e-03 |  |
|  | FREIJE (7) | 0.368 | 1.6e-03 |  |
|  | KAWAGUCHI (9) | 0.475 | 6.0e-03 |  |
|  | PAUGH (10) | 0.417 | 0.04 |  |
|  | | | | |
| **E2F1** | REMBRANDT (2, 3) | 0.245 | 1.85e-04 | 1 |

**Supplementary table 2 (Continuation)**

| **E2F6** | SUN (1) | 0.670 | 2.7e-11 | 5 |
| --- | --- | --- | --- | --- |
|  | REMBRANDT (2, 3) | 0.631 | 1.0e-26 |  |
|  | DONSON (4) | 0.514 | 3.2e-09 |  |
|  | FREIJE (7) | 0.642 | 1.6e-09 |  |
|  | KAWAGUCHI (9) | 0.567 | 7.2e-04 |  |

*EIF4EBP1* is co-expressed with each of the indicated transcription factor in the indicated cohorts. Co-expression levels were calculated with the Pearson correlation coefficient using the R^2^ genomic visualization platform (R^2^ AMC; http://r2.amc.nl).

**Supplementary table 3: Overview of the CNS and non-CNS tumor cohorts used for analyses depicted in figure 5.**

| **Tumor type** | **Cohort name** | **GEO ID or PUB med link** |
| --- | --- | --- |
| **Adult glioma** | Tumor Glioblastoma - Hegi - 84 | GSE7696 |
|  | Tumor Glioblastoma - Loeffler - 70 | GSE53733 |
|  | Tumor Glioma - French - 284 | GSE16011 |
|  | Tumor Glioma - Kawaguchi - 50 | GSE43378 |
|  | Tumor Glioma - Sun - 153 | GSE4290 |
|  | Tumor Glioma - Yan - 21 | GSE50774 |
|  | Tumor Glioma (CIC mutation status) - Gleize - 30 | Unknown |
|  | Tumor Brain (REMBRANDT study) - Madhavan - 550 | GSE108474 |
| **Breast cancer** | Tumor Breast - Black - 107 | GSE36771 |
|  | Tumor Breast - Bos - 204 | GSE12276 |
|  | Tumor Breast - Desmedt - 55 | GSE16391 |
|  | Tumor Breast - EXPO - 351 | GSE2109 |
|  | Tumor Breast - Iglehart - 123 | GSE5460 |
|  | Tumor Breast - Yu - 683 | GSE102484 |
|  | Tumor Breast (Anthracycline) - Sotiriou - 120 | GSE16446 |
|  | Tumor Breast (Chemotherapy) - Quiles - 61 | GSE28844 |
|  | Tumor Breast (HER2) - Concha - 66 | GSE29431 |
|  | Tumor Breast (MDC) - Bertucci - 266 | GSE21653 |
|  | Tumor Breast (mutation status) - Meijers-Heijboer - 155 | GSE27830 |
|  | Tumor Breast (TNBC) - Brown - 198 | GSE76124 |
| **Cancer of the gastrointestinal tract** | Tumor Colon - Marra - 32 | GSE8671 |
|  | Tumor Colon - Olschwang - 130 | GSE37892 |
|  | Tumor Colon (KRAS mut) - Hase - 59 | GSE92921 |
|  | Tumor Colon FOLFOX - Yagi - 83 | GSE28702 |
|  | Tumor Colon Rectum - EXPO - 38 | GSE2109 |
|  | Tumor Esophageal - Minashi - 40 | GSE32701 |
|  | Tumor Gastric - Tan - 192 | GSE15459 |
|  | Tumor Oral Cavity - Holsinger - 103 | GSE42743 |
| **Gynecologic cancer** | Tumor Endometrium - EXPO - 209 | GSE2109 |
|  | Tumor Ovarian - Anglesio - 90 | GSE2109 |
|  | Tumor Ovarian - Bowtell - 285 | GSE9891 |
|  | Tumor Ovarian - McDonald - 31 | GSE112798 |
|  | Tumor Ovarian (stroma) - McDonald - 45 | GSE38666 |
| **Leukemia** | Tumor ALL (T) - Meijerink - 124 | GSE26713 |
|  | Tumor ALL (T) - Pieters - 92 | GSE10609 |
|  | Tumor AML - Delwel - 460 | GSE6891 |
|  | Tumor AML CEBPA - Verhaak - 525 | GSE14468 |

**Supplementary table 3 (Continuation)**

| **Leukemia** | Tumor B-cell Lymphoma (High Grade) - Chan - 61 | GSE168422 |
| --- | --- | --- |
|  | Tumor B-cell non-Hodgkin Lymphoma (B-NHL) - Green - 290 | GSE132929 |
|  | Tumor CLL - Kueppers - 46 | GSE36907 |
|  | Tumor Lymphoma (PCNSL/PMLBCL) - Shipp - 26 | GSE61578 |
|  | Tumor MALT lymphoma - Du - 14 | GSE16024 |
|  | Tumor PCNSL Lymphoma - Sano - 34 | GSE34771 |
|  | Tumor T-cell lymphoma - Iqbala - 147 | GSE19069 |
| **Lung cancer** | Tumor Lung - Bild - 114 | GSE3141 |
|  | Tumor Lung - EXPO - 121 | GSE2109 |
|  | Tumor Lung - Peitsch - 150 | GSE43580 |
|  | Tumor Lung (NSCLC) - Chuang - 120 | GSE19804 |
|  | Tumor Lung (NSCLC) - Muley - 100 | GSE33532 |
|  | Tumor Non-small cell lung carcinoma - Plamadeala - 410 | GSE63074 |
| **Neuroblastoma** | Tumor Neuroblastic mixed - Delattre - 64 | GSE12460 |
|  | Tumor Neuroblastoma - Hiyama - 51 | GSE16237 |
|  | Tumor Neuroblastoma - Lastowska - 30 | GSE13136 |
|  | Tumor Neuroblastoma public - Versteeg - 88 | GSE16476 |
| **Pediatric brain cancer** | Tumor ATRT - Kool - 49 | GSE70678 |
|  | Tumor CNS-PNET - Kool - 182 | GSE73038 |
|  | Tumor CNS/PNET - Grundy - 24 | GSE19404 |
|  | Tumor Ependymoma - Donson - 19 | GSE16155 |
|  | Tumor Ependymoma - Gilbertson - 83 | 20639864 |
|  | Tumor Ependymoma - Hoffman - 65 | GSE50385 |
|  | Tumor Ependymoma - Pfister - 209 | GSE64415 |
|  | Tumor Glioma pediatric - Paugh - 53 | GSE19578 |
|  | Tumor Medulloblastoma - ATRT - Hsieh - 31 | GSE67851 |
|  | Tumor Medulloblastoma - Gilbertson - 76 | GSE37418 |
|  | Tumor Medulloblastoma - Pfister - 223 | 28726821 |
|  | Tumor Medulloblastoma Ependymoma - denBoer - 51 | GSE74195 |
|  | Tumor Medulloblastoma PLoS One - Kool - 62 | GSE10327 |
|  | Tumor Medulloblastoma public - Delattre - 57 | Unknown |
|  | Tumor Pilocytic Astrocytomas - Gutman - 41 | GSE5675 |
| **Sarcoma** | Tumor Ewing Sarcoma - Delattre - 117 | GSE34620 |
|  | Tumor Ewing Sarcoma - Francesconi - 37 | GSE12102 |
|  | Tumor Ewing Sarcoma - Surdez - 79 | GSE142162 |
|  | Tumor Osteosarcoma - Kobayashi - 27 | GSE14827 |
|  | Tumor Rhabdomyosarcoma - Barr - 58 | GSE66533 |

All cohorts were selected with the microarray mas5.0 - u133p2; GEO ID is indicated by GSE; Pub med link is indicated by a sequence of numbers.

**Supplementary table 4: Overview of the IDH status in the various glioma cohorts.**

| **Cohort** | **Reference** | **Accession no.** | **Number of patients** | **Diagnosis** | **IDH mutation status** |
| --- | --- | --- | --- | --- | --- |
| CGGA | (12) |  | n=85 | Primary glioblastoma | wildtype: n=74  mutant: n=11 |
| DONSON | (4) | GSE50161 | n=34 | Pediatric glioblastoma | Unknown |
| FREIJE | (7) | GSE4412 | n=71 | Glioma grade 4 | Unknown |
| FRENCH | (8) | GSE16011 | n=159 | Glioblastoma grade 4 | wildtype: n=59  mutant: n=33  Unknown: n=67 |
| FRENCH | (8) | GSE16011 | n= 42 | Glioma grade 2-4 | mutant: n=42 |
| HEGI | (5) | GSE7696 | n=70 | Glioblastoma | Unknown: n=70 |
| KAWAGUCHI | (9) | GSE43378 | n=32 | Glioblastoma | wildtype: n=31  mutant: n=1 |
| PAUGH | (10) | GSE26576 | n=25 | Pediatric glioblastoma  (Diffuse intrinsic pontine gliomas) | Unknown: n=25 |
| REMBRANDT | (2, 3) | GSE108474 | n=228 | Glioblastoma | not provided |
| SUN | (1) | GSE4290 | n=77 | Glioblastoma | not provided |
| TCGA | (6) |  | n=507 | Glioblastoma | wildtype: n=295  mutant: n=19  Unknown: n=193 |
| TUYSUZ | (11) | GSE90598 | n=16 | Glioblastoma | wildtype: n=12  mutant: n=4 |

**Supplementary table 5: List of siRNA sequences**

| **Target gene and siRNA name** | **SiRNA sequence** |
| --- | --- |
| Dharmacon | |
| Non-targeting | 5’- UAAGGCUAUGAAGAGAUAC -3’ |
|  | 5’- AUGUAUUGGCCUGUAUUAG -3’ |
|  | 5’- AUGAACGUGAAUUGCUCAA -3’ |
|  | 5’- UGGUUUACAUGUCGACUAA -3’ |
| ETS1 si2 | 5’- GGACCGUGCUGACCUCAAU -3’ |
| ETS1 si3 | 5’- GGAAUUACUCACUGAUAAA -3’ |
| MYBL2 si4 | 5’- UAACCGCACUGACCAGCAA -3’ |
| MYBL2 si5 | 5’- GUAACAGCCUCACGCCCAA -3’ |
| siPool Biotech | |
| Negative Control si Pool | 5’- UAGCGACUAAACACAUCAA -3’ |
|  | 5’- UAAGGCUAUGAAGAGAUAC -3’ |
|  | 5’- AUGUAUUGGCCUGUAUUAG -3’ |
|  | 5’- AUGAACGUGAAUUGCUCAA -3’ |
| E2F6 si Pool | 5’- TCACAAGTTAAGGAACTGC -3’ |
|  | 5’- TATTCTGTCAAACAGTACG -3’ |
|  | 5’- TCTGCTGGAGCTTTAACTG -3’ |
|  | 5’- TGATAGAGTCTTCTCTGGG -3’ |
|  | 5’- ACAAATAGACATCGATAGG -3’ |

**Supplementary table 5 (Continuation)**

| E2F6 si Pool | 5’- TCGATAGGTCCGTTGGTGC -3’ |
| --- | --- |
|  | 5’- TTTGGTAAAACCTTTGTTG -3’ |
|  | 5’- AAGAGGTCCCGACACCTTC -3’ |
|  | 5’- TCAAACAGCTGCTGAGCAC -3’ |
|  | 5’- AATCTGGTTTCTGCTGGAG -3’ |
|  | 5’- TTTGTTACTGGTCTGACCC -3’ |
|  | 5’- TCTGGGAGCTGGAACATCC -3’ |
|  | 5’- ATGAATGTCTTGATAGGTC -3’ |
|  | 5’- TCAGTTGCTTACTTCAAGC -3’ |
|  | 5’- TCTCCTTAAATATAGATGC -3’ |
|  | 5’- TGTTAACTCAAACAGCTGC -3’ |
|  | 5’- TGTTCAGACATTTATTGAG -3’ |
|  | 5’- TGAGAATCAAATTTGATGC -3’ |
|  | 5’- TTCTTTAAAAGCAATATTC -3’ |
|  | 5’- TCTGGATGAGTGCTCTCAG -3’ |
|  | 5’- TCTCAGATGAAGAGGTCCC -3’ |
|  | 5’- AATGCCATCAGTTGCTTAC -3’ |
|  | 5’- TAGGTCACATATGCTAGTC -3’ |
|  | 5’- AAGCAATTCTTCACTTTGC -3’ |
|  | 5’- ATCCTTAATTAACTCATCC -3’ |
|  | 5’- TTTAACTGCAATGACGATC -3’ |
|  | 5’- TTACTCAGTAATCTAAGTG -3’ |
|  | 5’- ATGCTAGTCTTTCATTTTC -3’ |
|  | 5’- TTATTGAGCACTTCTTAAG -3’ |
|  | 5’- AATGACGATCTGTTCATGG -3’ |

**Supplementary table 6: List of RT-qPCR primers**

| **Primer** | **Sequence** |
| --- | --- |
| 4EBP1 | FW: 5’-AGCCCTTCCAGTGATGAGC-3’  RV: 5’-TGTCCATCTCAAACTGTGACTCTT-3’ |
| E2F6 | FW: 5’-CGTTTTGATGTATCGCTGGTTTAT-3’  RV: 5’- TGCAACCTTGTTTAAGTCAAGAATACC-3’ |
| ETS1 | FW: 5’-AGTGGTGAGGCAAGGACCTA-3’  RV: 5’- ATCCCAAAAGGGGTAGCAAG-3’  (13) |
| GusB | FW: 5’-GTTTTTGATCCAGACCCAGATG-3’  RV: 5’-GCCCATTATTCAGAGCGAGTA-3’ |
| MYBL2 | FW: 5’-ACCTCCCTGAGGAACCATCT-3’  RV: 5’-AGGACTTGCTGCTGATGTGA-3’  (14) |
| PPIA | FW: 5’-TTATTTGGGTTGCTCCCTTC-3’  RV: 5’-AAGTGTGCCAAATCTGCAAG-3’ |
| $\text{β}$-actin | FW: 5’-TCCCCCAACTTGAGATGTATG-3’  RV: 5’-ACTGGTCTCAAGTCAGTGTACAGG-3’ |

**Supplementary table 7: List of antibodies**

| **Antibody** | **Company** | **Catalog number** |
| --- | --- | --- |
| 4EBP1 (53H11) | Cell signaling, Cambridge, UK | # 9644S |
| E2F1 | Cell signaling | # 3742S |
| E2F6 | Abcam, Cambridge, UK | # EPR11201 |
| eEF2 | Cell signaling | # 2332S |
| ETS1 (D808A) | Cell signaling | # 14069S |
| FOXM1 (D3F2B) | Cell signaling | # 20459S |
| GAPDH (14C10) | Cell signaling | # 2118S |
| HIF-1alpha (D2U3T) | Cell signaling | # 14179S |
| IRDye® 800CW Goat anti-Mouse IgG Secondary Antibody | LI-COR Bioscience, Bad Homburg, Germany | # 925-32210 |
| IRDye® 800CW Goat anti-Rabbit IgG Secondary Antibody | LI-COR Bioscience | # 925-32211 |
| JUN (60A8) | Cell signaling | # 9165L |
| MYBL2 | Abcam | # ab12296 |
| VINCULIN | Cell signaling | # 4650S |
| $\text{β}$-ACTIN | Sigma Aldrich, St Louis, USA | # A2228 |

**References**

1. Sun L, Hui AM, Su Q, Vortmeyer A, Kotliarov Y, Pastorino S, et al. Neuronal and glioma-derived stem cell factor induces angiogenesis within the brain. Cancer Cell. 2006;9(4):287-300.

2. Gusev Y, Bhuvaneshwar K, Song L, Zenklusen JC, Fine H, Madhavan S. The REMBRANDT study, a large collection of genomic data from brain cancer patients. Sci Data. 2018;5:180158.

3. Madhavan S, Zenklusen JC, Kotliarov Y, Sahni H, Fine HA, Buetow K. Rembrandt: helping personalized medicine become a reality through integrative translational research. Mol Cancer Res. 2009;7(2):157-67.

4. Griesinger AM, Birks DK, Donson AM, Amani V, Hoffman LM, Waziri A, et al. Characterization of distinct immunophenotypes across pediatric brain tumor types. Journal of immunology. 2013;191(9):4880-8.

5. Murat A, Migliavacca E, Gorlia T, Lambiv WL, Shay T, Hamou MF, et al. Stem cell-related "self-renewal" signature and high epidermal growth factor receptor expression associated with resistance to concomitant chemoradiotherapy in glioblastoma. J Clin Oncol. 2008;26(18):3015-24.

6. Cancer Genome Atlas Research N, Weinstein JN, Collisson EA, Mills GB, Shaw KR, Ozenberger BA, et al. The Cancer Genome Atlas Pan-Cancer analysis project. Nat Genet. 2013;45(10):1113-20.

7. Freije WA, Castro-Vargas FE, Fang Z, Horvath S, Cloughesy T, Liau LM, et al. Gene expression profiling of gliomas strongly predicts survival. Cancer research. 2004;64(18):6503-10.

8. Gravendeel LA, Kouwenhoven MC, Gevaert O, de Rooi JJ, Stubbs AP, Duijm JE, et al. Intrinsic gene expression profiles of gliomas are a better predictor of survival than histology. Cancer research. 2009;69(23):9065-72.

9. Kawaguchi A, Yajima N, Tsuchiya N, Homma J, Sano M, Natsumeda M, et al. Gene expression signature-based prognostic risk score in patients with glioblastoma. Cancer Sci. 2013;104(9):1205-10.

10. Paugh BS, Broniscer A, Qu C, Miller CP, Zhang J, Tatevossian RG, et al. Genome-wide analyses identify recurrent amplifications of receptor tyrosine kinases and cell-cycle regulatory genes in diffuse intrinsic pontine glioma. J Clin Oncol. 2011;29(30):3999-4006.

11. Gulluoglu S, Tuysuz EC, Sahin M, Kuskucu A, Kaan Yaltirik C, Ture U, et al. Simultaneous miRNA and mRNA transcriptome profiling of glioblastoma samples reveals a novel set of OncomiR candidates and their target genes. Brain Res. 2018;1700:199-210.

12. Zhao Z, Zhang KN, Wang Q, Li G, Zeng F, Zhang Y, et al. Chinese Glioma Genome Atlas (CGGA): A Comprehensive Resource with Functional Genomic Data from Chinese Glioma Patients. Genomics Proteomics Bioinformatics. 2021;19(1):1-12.

13. Khanna A, Mahalingam K, Chakrabarti D, Periyasamy G. Ets-1 expression and gemcitabine chemoresistance in pancreatic cancer cells. Cell Mol Biol Lett. 2011;16(1):101-13.

14. Tripathi V, Shen Z, Chakraborty A, Giri S, Freier SM, Wu X, et al. Long noncoding RNA MALAT1 controls cell cycle progression by regulating the expression of oncogenic transcription factor B-MYB. PLoS Genet. 2013;9(3):e1003368.
